# The Relationship of the Anti-Oxidant Bilirubin with Free Thyroxine Is Modified by Insulin Resistance in Euthyroid Subjects

**DOI:** 10.1371/journal.pone.0090886

**Published:** 2014-03-03

**Authors:** Petronella E. Deetman, Stephan J. L. Bakker, Arjan J. Kwakernaak, Gerjan Navis, Robin P. F. Dullaart, on behalf of the PREVEND Study Group

**Affiliations:** Department of Internal Medicine, University of Groningen, University Medical Center Groningen, The Netherlands; University of Warwick – Medical School, United Kingdom

## Abstract

**Background:**

The strong anti-oxidative properties of bilirubin largely explain its cardioprotective effects. Insulin resistance is featured by low circulating bilirubin. Thyroid hormone affects both bilirubin generation and its biliary transport, but it is unknown whether circulating bilirubin is associated with thyroid function in euthyroid subjects. Aim is to determine relationships of bilirubin with TSH, free T_4_ and free T_3_ in euthyroid subjects without type 2 diabetes mellitus (T2DM), and to assess whether such a relationship would be modified by the degree of insulin resistance.

**Methods:**

Total bilirubin, TSH, free T_4_, free T_3_, glucose, insulin, lipids and transaminases were measured in 1854 fasting euthyroid subjects without T2DM, recruited from the general population (PREVEND cohort). Insulin resistance was assessed by homeostasis model assessment.

**Results:**

Bilirubin was positively related to free T_4_ (β = 0.116, *P*<0.001) and free T_3_ (β = 0.078, *P* = 0.001), but bilirubin was unrelated to TSH. The relationship of bilirubin with free T_4_ was modified by insulin resistance with a larger effect in more insulin resistant individuals (adjusted for age and sex: β = 0.043, *P* = 0.056 for interaction; additionally adjusted for smoking, alcohol intake, transaminases and total cholesterol (β = 0.044, *P* = 0.044 for interaction). The association of bilirubin with free T_4_ was also modified by high density lipoprotein cholesterol (age- and sex-adjusted: β = 0.040, *P* = 0.072).

**Conclusions:**

Low bilirubin relates to low free T_4_ in euthyroid non-diabetic subjects. Low normal free T_4_ may particularly confer low bilirubin in more insulin resistant individuals.

## Introduction

It is increasingly appreciated that endogenous bilirubin has strong anti-oxidative properties, which are attributed to its ability to scavenge peroxyl radicals and to inhibit low density lipoprotein (LDL) oxidation [1]. Hence, the concept is emerging that bilirubin is involved in the pathogenesis of cardiometabolic disorders in which oxidative-stress is considered to play an important role [1]–[3]. In this line, low circulating levels of bilirubin levels have been documented to be associated with increased severity of atherosclerosis [4] and higher risk of lower limb amputation [5]. Low levels of circulating bilirubin have also been associated with increased cardiovascular and all-cause mortality in men [6]. In addition, intima media thickness, an established marker of subclinical atherosclerosis, is smaller in subjects with isolated hyperbilirubinemia [7]. Conversely, increased intima media thickness relates to low bilirubin in middle-aged subjects [8].

The importance of bilirubin for the development of atherosclerotic cardiovascular diseases underscores the relevance to delineate the metabolic factors that affect its metabolism in more detail. Thyroid hormones stimulate heme oxygenase-1 activity (HO-1), which is the main enzyme responsible for bilirubin production [9], [10]. Furthermore, thyroid hormones downregulate the enzymatic activity of uridine 5′-diphospho-glucuronosyltransferase (UDP-GT), which stimulates bilirubin conjugation, thereby facilitating bilirubin excretion [11], [12]. In agreement with the hypothesis that thyroid function represents a clinically relevant determinant of serum bilirubin metabolism, we have recently shown that low free T_4_ levels confer decreased bilirubin levels in euthyroid patients with type 2 diabetes mellitus (T2DM) [13]. Of further interest, insulin resistance and the metabolic syndrome (MetS) are not only featured by low bilirubin levels, but also by low free T_4_ [14], [15]. In extension thereof, it may be hypothesized that a possible relationship of bilirubin with thyroid function among euthyroid subjects is influenced by insulin resistance.

Against this background, the present study was initiated to test whether low plasma bilirubin is related to a lower thyroid functional status in euthyroid non-diabetic subjects recruited from the general population. Second, we determined the extent to which such a relationship is modified by the degree of insulin resistance and MetS components.

## Methods

### Subjects

The population used for this study consisted of a random subset of participants of the PREVEND (Prevention of Renal and Vascular End Stage Disease) study, which are inhabitants, aged 28–75 yr, of the city of Groningen, The Netherlands. The protocol of this study has been described elsewhere [16], [17]. The medical ethics committee of the University Medical Center Groningen approved the study, and all participants gave written informed consent. A health questionnaire indicated that the participants had no history of liver disease.

For the current analysis, we excluded subjects not being euthyroid, subjects using thyroid hormones, anti-thyroid drugs and amiodarone, subjects with diabetes mellitus (as indicated by self-reported questionnaire, a physician diagnosis of diabetes, the use of oral glucose-lowering medication and/or elevated plasma glucose), as well as subjects in whom blood was not taken in the fasting state. Euthyroidism was defined as TSH, free T_4_ and free T_3_ levels within the reference range as provided by the manufacturer (see Laboratory analyses). We additionally excluded subjects with positive anti-thyroid peroxidase auto-antibodies (cut-off value: see Laboratory analyses). Applying these selection criteria, 1854 subjects were eligible for the current analyses.

Patient characteristics, including age, sex, alcohol use, smoking status, body mass index (BMI), systolic and diastolic blood pressure, and waist circumference were obtained. Blood was drawn after an overnight fasting period for measurement of free T_4_, free T_3_, TSH, bilirubin, glucose, insulin, total cholesterol, high density lipoprotein (HDL) cholesterol, triglycerides, aspartate aminotransferase (AST), and alanine aminotransferase (ALT).

Body mass index was defined as weight (kg) by height (m) squared. Alcohol consumption was recorded with one drink being assumed to contain 10 grams of alcohol. Insulin resistance was estimated using the Homeostasis Model Assessment-Insulin Resistance (HOMA-IR): glucose (mmol/L) × insulin (mU/L)/22.5 [18]. Three or more of the following criteria were required for categorization of subjects with MetS: waist circumference >102 cm for men and >88 cm for women, hypertension (blood pressure ≥130/85 mmHg or use of anti-hypertensive drugs), fasting triglycerides ≥1.70 mmol/L, fasting glucose ≥5.6 mmol/L, and HDL cholesterol <1.03 mmol/L for men and <1.29 mmol/L for women [19].

### Laboratory analyses

Heparinized plasma and serum samples were stored at −80°C until analyses. Serum TSH (Architect; Abbott Laboratories, Abbott Park, IL, USA; reference range 0.35–4.94 mU/L), free T_4_ (AxSYM; Abbott Laboratories, Abbott Park, IL, USA; reference range 9.14–23.81 pmol/L) and free T_3_ (AxSYM; Abbott Laboratories, Abbott Park, IL, USA; reference range; 2.23–5.35 pmol/L) were measured by microparticle enzyme immunoassay. Anti-thyroid peroxidase autoantibodies were determined using commercially available automated enzyme linked immunoassays (Abbott Laboratories, Abbott Park, IL, USA; kit number 5F57). Anti-thyroid peroxidase autoantibodies were considered positive using a cutoff value as indicated by the supplier (≥12 kU/L). Plasma total bilirubin was measured by a colorimetric assay (2,4-dicholoraniline reaction; Merck MEGA, Darmstadt, Germany). In healthy subjects, bilirubin is most abundantly present in serum in its unconjugated form [20]. In a validation experiment (n = 80), a strong correlation between total bilirubin and unconjugated bilirubin (Spearman's r = 0.92, *P*<0.001), as well as between total bilirubin and conjugated direct bilirubin (Spearman's r = 0.82, *P*<0.001) was observed. For the present study we only used total bilirubin in keeping with other reports [21]–[23]. Serum ALT and AST were measured with pyridoxal phosphate activation (Merck MEGA, Darmstadt, Germany). Serum total cholesterol and plasma glucose were measured using Kodak Ektachem dry chemistry (Eastman Kodak, Rochester, NY, USA). Serum triglycerides were measured enzymatically. HDL cholesterol was measured with a homogeneous method (direct HDL, AEROSET system; Abbott Laboratories, Abbott Park, IL, USA; no. 7D67). Insulin was measured by microparticle enzyme immunoassay (AxSYM; Abbott Laboratories, Abbott Park, IL, USA).

### Statistical analyses

Data analyses were performed using *SPSS* (version 20.0, SPSS Inc. Chicago, IL, USA). Normally distributed data are given as mean ± standard deviation (SD) and non-parametrically distributed data are presented as median (interquartile range, IQR). Categorical variables are given as percentages. Differences in bilirubin concentration between sexes were determined by Mann-Whitney U-test. Characteristics of the study population are presented according to sex-stratified tertiles of bilirubin. Univariable linear regression analysis was used to test for linear trends across tertiles of bilirubin. Multivariable linear regression analyses were used to determine the extent to which bilirubin is related to thyroid function, components of the metabolic syndrome, insulin, HOMA-IR and transaminases. To this end, logarithmically transformed values of bilirubin, glucose, insulin, HOMA-IR, triglycerides, TSH and transaminases were used. Multivariable models were all age- and sex-adjusted. Before calculating interaction terms, the continuous variable of interest were centered to the mean by subtracting the group mean value from individual values. This was done in order to avoid multicollinearity [24], [25]. Interaction terms were considered statistically significant at *P*-values <0.10, as proposed by Selvin [26] and recommended by the Food and Drug Administration authorities [27]. Otherwise, two-sided *P*-values <0.05 were considered significant.

## Results

A total of 1854 subjects (age 47±13, 50% men) participated in this study. Median bilirubin concentration was 8 (6–10) µmol/L in men and 6 (5–8) µmol/L in women (*P*<0.001). Clinical and laboratory characteristics of the study population are, therefore, shown according to sex-stratified tertiles of bilirubin (Table 1). Angiotensin converting enzyme inhibitors (ACEi) or angiotensin receptor blockers (ARB's) were used by 56 subjects (3%); 46 subjects (3%) used lipid lowering drugs (mainly statins).

**Table 1:** Clinical characteristics, glucose, insulin, insulin resistance, lipids, transaminases, and thyroid hormones in 1854 subjects.

	*Sex-stratified tertiles of bilirubin*				
	*1*	*2*	*3*		
	*Men (n = 335)*	*Men (n = 324)*	*Men (n = 263)*		
	*<7 μmol/L*	*7–9 μmol/L*	*>9 μmol/L*		
	*Women (n = 354)*	*Women (n = 274)*	*Women (n = 291)*		
	*<6 μmol/L*	*6–7 μmol/L*	*>7 μmol/L*	*β*	*P-value*
Age (years)	48±12	48±13	46±13	−0.037	0.109
BMI (kg/m^2^)	26.5±4.5	25.7±4.4	25.1±3.9	−0.114	<0.001
Alcohol				0.077	0.001
<10 gram per day (%)	76	69	72		
≥10 gram per day (%)	24	31	28		
Current smoker (%)	44	36	27	−0.163	<0.001
Waist circumference in men (cm)	95±12	92±11	92±12	−0.109	0.001
Waist circumference in women (cm)	83±13	82±13	80±11	−0.125	<0.001
Systolic blood pressure (mmHg)	129±21	129±19	126±20	0.022	0.338
Diastolic blood pressure (mmHg)	74±9	74±9	73±10	0.012	0.595
Glucose (mmol/L)	4.4 (4.0–4.9)	4.4 (4.0–4.8)	4.2 (3.9–4.6)	−0.028	0.234
Insulin (mU/L)	9.0 (6.1–13.2)	7.6 (5.5–11.1)	7.1 (5.1–10.0)	−0.133	<0.001
HOMA-IR	1.74 (1.15–2.76)	1.47 (1.03–2.26)	1.33 (0.92–1.94)	−0.122	<0.001
Total cholesterol (mmol/L)	5.82±1.16	5.60±1.16	5.41±1.08	−0.113	<0.001
HDL cholesterol (mmol/L)	1.32±0.39	1.35±0.39	1.43±0.43	0.031	0.189
Triglycerides (mmol/L)	1.24 (0.89–1.87)	1.12 (0.84–1.56)	1.01 (0.74–1.43)	−0.121	<0.001
Metabolic syndrome (%)	25	17	13	−0.101	0.001
TSH (mU/L)	1.34 (0.98–1.87)	1.28 (0.94–1.82)	1.37 (1.00–1.85)	−0.033	0.152
Free T_4_ (pmol/L)	12.66±1.69	13.08±1.79	13.04±1.77	0.116	<0.001
Free T_3_ (pmol/L)	3.69±0.62	3.72±0.61	3.80±0.62	0.078	0.001
AST (U/L)	24 (21–28)	24 (21–29)	25 (21–29)	0.154	<0.001
ALT (U/L)	20 (15–28)	20 (16–28)	20 (15–29)	0.115	<0.001

Data in mean ± SD or in median (interquartile range). BMI, body mass index; HOMA-IR, homeostasis model assessment-insulin resistance; HDL, high density lipoprotein; ALT, alanine aminotransferase; AST, aspartate aminotransferase; β, standardized regression coefficient. *P*-values for linear trend are shown. Bilirubin, glucose, insulin, HOMA-IR, triglycerides, TSH, AST and ALT were log transformed.

In univariable analyses, bilirubin was inversely related to age, BMI, smoking status, waist circumference, insulin, HOMA-IR, total cholesterol, triglycerides, and the presence of metabolic syndrome (Table 1). We also found positive relationships of bilirubin with free T_4_ and free T_3_, alcohol use, ALT, and AST. Bilirubin was not associated with blood pressure, glucose, HDL cholesterol and TSH. There were no interactions of sex with free T_4_, free T_3_ and TSH on bilirubin (*P*>0.29 for all; data not shown). In age- and sex-adjusted linear regression analyses (Table 2), bilirubin was positively associated with free T_4_, but there were no significant associations of bilirubin with free T_3_ and TSH. Bilirubin was inversely associated with diastolic blood pressure, waist circumference, glucose, insulin, HOMA-IR, total cholesterol, HDL cholesterol and triglycerides in age- and sex-adjusted analyses (Table 2).

**Table 2:** Age- and sex-adjusted linear regression analyses demonstrating relationships of bilirubin with thyroid hormones, components of the metabolic syndrome, insulin, insulin resistance and total cholesterol.

	*Total bilirubin (μmol/L)*	
	*β*	*P-value*
TSH (mU/L)	−0.015	0.510
Free T_4_ (pmol/L)	0.086	<0.001
Free T_3_ (pmol/L)	0.033	0.150
Systolic blood pressure (mmHg)	−0.028	0.296
Diastolic blood pressure (mmHg)	−0.058	0.026
Waist circumference (cm)	−0.116	<0.001
Glucose (mmol/L)	−0.055	0.022
Insulin (mU/L)	−0.144	<0.001
HOMA-IR	−0.143	<0.001
Total cholesterol (mmol/L)	−0.121	<0.001
HDL cholesterol (mmol/L)	0.154	<0.001
Triglycerides (mmol/L)	−0.176	<0.001

HDL, high density lipoprotein; HOMA-IR, homeostasis model assessment-insulin resistance; β, standardized regression coefficient. Bilirubin, TSH, glucose, insulin and HOMA-IR and triglycerides were log transformed.

**Figure 1:**
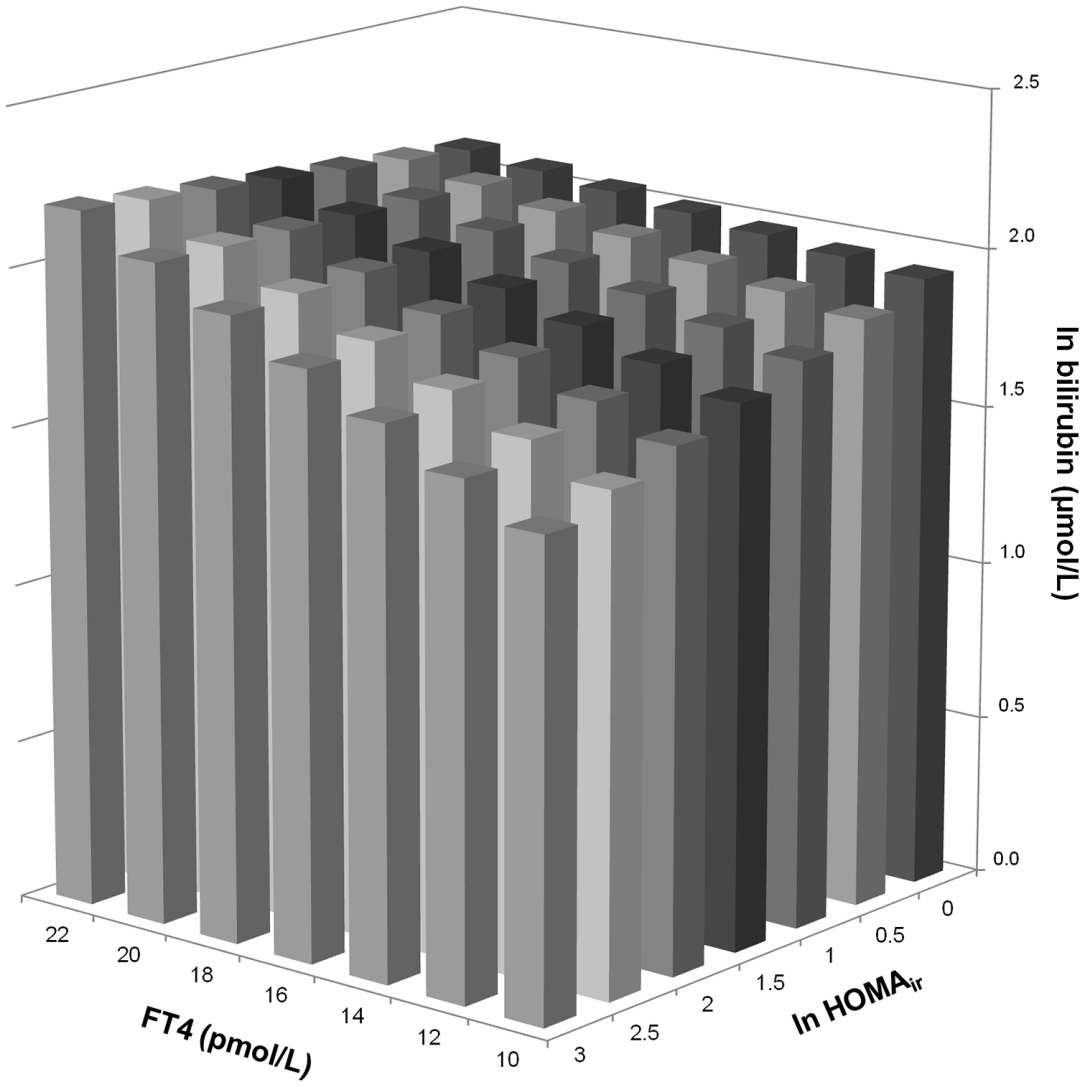
Graphical presentation of the interaction of free T_4_ with insulin resistance on bilirubin. The standardized regression coefficients of the interaction term obtained by multivariable linear regression analysis as shown in Table 3, model 3 is used.

We then tested whether the relationship of bilirubin with free T_4_ was modified by HOMA-IR, fasting insulin, individual MetS components, and total cholesterol. The relationship of bilirubin with free T_4_ was significantly modified by HOMA-IR (β = 0.043, *P* = 0.056 for interaction; Table 3) and by plasma insulin (β = 0.040, *P* = 0.072 for interaction; Table S1). The effect-modification of free T_4_ by HOMA-IR was independent of potential confounding factors including smoking, alcohol use ≥10 gram/day, AST, ALT and total cholesterol (β = 0.044, *P* = 0.044 for interaction; Table 3). Figure 1 provides a graphical presentation of the modification of the effect of free T_4_ on bilirubin by HOMA-IR. As shown in Table S1, there were no significant modifications of the effect of free T_4_ on bilirubin by systolic blood pressure, diastolic blood pressure, waist circumference, glucose, total cholesterol, or triglycerides in age- and sex-adjusted analyses (*P*>0.27 for all), but there was a significant modification of the effect of free T_4_ on bilirubin by HDL cholesterol (β = 0.040, *P* = 0.072). In addition, there were no significant modifications of a potential effect of free T_3_ on bilirubin by HOMA-IR, fasting insulin, components of the metabolic syndrome, and total cholesterol (*P*>0.32 for all; data not shown).

**Table 3:** Multivariable linear regression models demonstrating the interaction between free T_4_ and insulin resistance on bilirubin.

	*Model 1*		*Model 2*		*Model 3*	
	*β*	*P-value*	*β*	*P-value*	*β*	*P-value*
Age (years)	−0.030	0.196	−0.031	0.189	−0.004	0.864
Sex (men/women)	−0.250	<0.001	−0.251	<0.001	−0.213	<0.001
Free T_4_ (pmol/L)	0.069	0.002	0.072	0.002	0.084	<0.001
HOMA-IR	−0.134	<0.001	−0.134	<0.001	−0.158	<0.001
Free T_4_*HOMA-IR			0.043	0.056	0.044	0.044
Current smoker (yes/no)					−0.184	<0.001
Alcohol intake (≥10 gram/day)					0.026	0.250
Total cholesterol (mmol/L)					−0.101	<0.001
AST (U/L)					0.097	0.003
ALT (U/L)					0.015	0.675

HOMA-IR, Homeostasis model assessment-insulin resistance; AST, Aspartate aminotransferase; ALT, Alanine aminotransferase; β, standardized regression coefficient. Bilirubin, HOMA-IR, AST and ALT were log transformed.

Secondary analyses were performed after exclusion of subjects using lipid lowering drugs, ACEi and ARB's. In the remaining subjects (n = 1759), there was again an age- and sex-adjusted positive relationship of bilirubin with free T_4_ (β = 0.094, *P<*0.001). Furthermore, the interaction of free T_4_ with HOMA-IR on bilirubin was also significant in these analyses (β = 0.050, *P* = 0.030 for interaction), and remained significant after further adjustment for alcohol intake, transaminases and total cholesterol (β = 0.056, *P* = 0.013 for interaction).

## Discussion

To our knowledge, this is the first report on an independent positive relationship of total bilirubin with free T_4_ in a large group of euthyroid, non-diabetic individuals recruited from the general population. Of note, multivariable linear regression analyses demonstrated a significant positive modification of the effect of free T_4_ on bilirubin by insulin resistance as quantified by HOMA-IR. This effect-modification remained essentially unaltered after controlling for potential confounders, including smoking, alcohol, transaminases, and total cholesterol. Our results, therefore, are in concert with the hypothesis that low-normal thyroid function may confer lower circulating bilirubin levels, especially in insulin resistant individuals. In addition, the effect of free T_4_ on bilirubin was modified by the HDL cholesterol concentration.

We recently documented a positive relationship of circulating levels of bilirubin with free T_4_ in euthyroid T2DM subjects [13]. In that report, bilirubin was not significantly correlated with free T_4_ in non-diabetic subjects, possibly due to the limited number of participants. Moreover, it could not be determined what was the driving force behind this association, i.e. insulin resistance or hyperglycemia resulting from β-cell dysfunction. Our previous findings [13], therefore, endorsed our main rationale to investigate the association between bilirubin and free T_4_ in a large group of non-diabetic subjects. In the current study, we found bilirubin to be more strongly associated with HOMA-IR and insulin than with glucose in age- and sex-adjusted analysis. Furthermore, a positive modification of the effect of free T_4_ on bilirubin by HOMA-IR was observed in such a way that the effect of free T_4_ on bilirubin was most pronounced in the most insulin resistant subjects. This effect modification was not observed with plasma glucose, raising the possibility that insulin resistance rather than hyperglycemia *per se* could represent a mechanism linking low bilirubin to low normal thyroid function. In view of the strong anti-oxidative properties of the HDL fraction [28], and the modification of HDL anti-oxidative capacity by thyroid function [29], it is also of potential relevance that the effect of free T_4_ on bilirubin was modified by HDL cholesterol.

The interaction of free T_4_ with insulin resistance on bilirubin may have pathophysiological relevance since lower thyroid functional status, impaired insulin sensitivity, and low bilirubin are all characterized by enhanced oxidative stress [2], [30]–[32]. The positive relationship of bilirubin with free T_4_ may at least in part be explained by effects of thyroid function on bilirubin production, given the stimulatory effect of thyroid hormone on HO-1 expression, and the inhibitory effect on UDP-glucuronosyltransferase [10]–[12]. HO-1 expression is stimulated by insulin *in vitro* [33], [34]. Furthermore, plasma levels of HO-1 are elevated in subjects with pre-diabetes and are strongly correlated with HOMA-IR [35]. Taken these findings together, it is plausible to hypothesize that effects of thyroid hormone on HO-1 expression could be more prominent in hyperinsulinemic and more insulin resistant individuals. On the other hand, it is obvious that stimulatory effects of insulin on HO-1 expression alone cannot explain the lower bilirubin levels in insulin resistant and MetS subjects [2]. Although little explored, insulin could also affect bilirubin metabolism, since insulin deficiency may result in enhanced UDP-glucuronyltransferase activity and bilirubin excretion [36]. Further study is required to more precisely delineate the mechanisms responsible for the alleged effects of thyroid functional status on bilirubin metabolism. Moreover, it remains to be established why bilirubin was related to free T_4_ (and in univariable analysis also to free T_3_) but not to the TSH level, extending our previous report showing relationships of plasma lipids with free thyroid hormone levels rather than with TSH [13].

Several other methodological issues and limitations of the present study warrant consideration. First, euthyroidism was strictly defined as levels of free T_4_, free T_3_ and TSH within the assay-specific reference range as provided by the manufacturer. We also excluded subjects with positive anti-thyroid peroxidase auto-antibodies. This was done to reduce possible bias in the relationship of free thyroid hormone levels with TSH in subjects with very early stages of autoimmune thyroid dysfunction as much as possible. Second, we performed a cross-sectional study. Thus, cause-effect relationships cannot be established with certainty. However, bilirubin has been shown not to influence the set-point of the pituitary-thyroid axis [37], strongly suggesting that low bilirubin levels by themselves are unlikely to lower thyroid function. Third, statin treatment has been reported to decrease bilirubin levels [38], and to increase plasma glucose [39], whereas ACEi or ARB's are likely to inhibit oxidative stress [40] and to improve insulin sensitivity [41]. In primary analyses, we did not exclude subjects using lipid lowering drugs or individuals using ACEi or ARB's. Instead, we carried out a secondary analysis after exclusion of subjects using these medications. This secondary analysis showed an essentially unaltered relationship of bilirubin with free T_4_ and a similar interaction of free T_4_ with HOMA-IR on bilirubin.

In conclusion, the current study shows an independent relationship of low bilirubin with low free T_4_ in euthyroid subjects. Low normal free T_4_ may particularly confer low bilirubin in more insulin resistant individuals. Since bilirubin is a potent endogenous anti-oxidant, it is plausible to speculate that low normal thyroid functional status could enhance atherosclerosis susceptibility in the context of insulin resistance.

## Supporting Information

Table S1 **Multivariable linear regression models demonstrating interactions of free T_4_ with metabolic syndrome components on bilirubin.**
(DOCX)
