# Supplementary material for: The Relationship of the Anti-Oxidant Bilirubin with Free Thyroxine Is Modified by Insulin Resistance in Euthyroid Subjects
Source: PLoS One. 2014 Mar 3;9(3):e90886. doi: 10.1371/journal.pone.0090886 (PMC3940953; doi:10.1371/journal.pone.0090886)
Supplement: Table S1 — Multivariable linear regression models demonstrating interactions of free T4 with metabolic syndrome components on bilirubin. (DOCX) [file pone.0090886.s001.docx]

**Table S1.** Multivariable linear regression models demonstrating interactions of free T_4_ with metabolic syndrome components on bilirubin

|  | Model 1 | | Model 2 | |
| --- | --- | --- | --- | --- |
|  | β | *P-*value | β | *P-*value |
| **A** | | | | |
| Free T_4_ (pmol/L) | 0.086 | <0.001 | 0.086 | <0.001 |
| Systolic blood pressure (mmHg) | -0.028 | 0.302 | -0.028 | 0.302 |
| Free T_4_ * systolic blood pressure |  |  | -0.0002 | 0.994 |
| **B** | | | | |
| Free T_4_ (pmol/L) | 0.085 | <0.001 | 0.085 | <0.001 |
| Diastolic blood pressure (mmHg) | -0.056 | 0.031 | -0.056 | 0.031 |
| Free T_4_* diastolic blood pressure |  |  | -0.006 | 0.787 |
| **C** |  |  |  |  |
| Free T_4_ (pmol/L) | 0.077 | 0.001 | 0.077 | 0.001 |
| Waist circumference (cm) | -0.107 | <0.001 | -0.108 | <0.001 |
| Free T_4_* waist circumference (cm) |  |  | -0.004 | 0.860 |
| **D** | | | | |
| Free T_4_ (pmol/L) | 0.083 | <0.001 | 0.084 | <0.001 |
| Glucose (mmol/L) | -0.050 | 0.039 | -0.053 | 0.029 |
| Free T_4_ * glucose |  |  | 0.025 | 0.266 |
| **E** | | | | |
| Free T_4_ (pmol/L) | 0.069 | 0.002 | 0.071 | 0.002 |
| Insulin (mU/L) | -0.136 | <0.001 | -0.135 | <0.001 |
| Free T_4_ * insulin |  |  | 0.040 | 0.072 |
| **F** | | | | |
| Free T_4_ (pmol/L) | 0.080 | <0.001 | 0.081 | <0.001 |
| Total cholesterol (mmol/L) | -0.116 | <0.001 | -0.117 | <0.001 |
| Free T_4_* total cholesterol |  |  | 0.007 | 0.747 |
| **G** | | | | |
| Free T_4_ (pmol/L) | 0.079 | <0.001 | 0.077 | 0.001 |
| HDL cholesterol (mmol/L) | 0.149 | <0.001 | 0.151 | <0.001 |
| Free T_4_ * HDL cholesterol |  |  | 0.040 | 0.072 |
| **H** | | | | |
| Free T_4_ (pmol/L) | 0.071 | 0.002 | 0.116 | 0.040 |
| Triglycerides (mmol/L) | -0.169 | <0.001 | -0.169 | <0.001 |
| Free T_4_* triglycerides |  |  | 0.050 | 0.381 |

All models are age and sex adjusted. HDL, high density lipoprotein; β, standardized regression coefficient. Glucose, insulin and triglycerides were log transformed.
